# Defining the molecular signatures of Achilles tendinopathy and anterior cruciate ligament ruptures: A whole-exome sequencing approach

**DOI:** 10.1371/journal.pone.0205860

**Published:** 2018-10-25

**Authors:** Andrea Gibbon, Colleen J. Saunders, Malcolm Collins, Junaid Gamieldien, Alison V. September

**Affiliations:** 1 Division of Exercise Science and Sports Medicine, Department of Human Biology, Faculty of Health Sciences, University of Cape Town, Cape Town, South Africa; 2 South African National Bioinformatics Institute/SA MRC Unit for Bioinformatics Capacity Development, University of the Western Cape, Bellville, Cape Town, South Africa; 3 Division of Emergency Medicine, Department of Surgery, Faculty of Health Sciences, University of Cape Town, Cape Town, South Africa; Queen Mary University of London, UNITED KINGDOM

## Abstract

Musculoskeletal soft tissue injuries are complex phenotypes with genetics being one of many proposed risk factors. Case-control association studies using the candidate gene approach have predominately been used to identify risk loci for these injuries. However, the ability to identify all risk conferring variants using this approach alone is unlikely. Therefore, this study aimed to further define the genetic profile of these injuries using an integrated omics approach involving whole exome sequencing and a customised analyses pipeline. The exomes of ten exemplar asymptomatic controls and ten exemplar cases with Achilles tendinopathy were individually sequenced using a platform that included the coverage of the untranslated regions and miRBase miRNA genes. Approximately 200 000 variants were identified in the sequenced samples. Previous research was used to guide a targeted analysis of the genes encoding the tenascin-C (*TNC)* glycoprotein and the α1 chain of type XXVII collagen (*COL27A1)* located on chromosome 9. Selection of variants within these genes were; however, not predetermined but based on a tiered filtering strategy. Four variants in *TNC* (rs1061494, rs1138545, rs2104772 and rs1061495) and three variants in the upstream *COL27A1* gene (rs2567706, rs2241671 and rs2567705) were genotyped in larger Achilles tendinopathy and anterior cruciate ligament (ACL) rupture sample groups. The CC genotype of *TNC* rs1061494 (C/T) was associated with the risk of Achilles tendinopathy (*p* = 0.018, OR: 2.5 95% CI: 1.2–5.1). Furthermore, the AA genotype of the *TNC* rs2104772 (A/T) variant was significantly associated with ACL ruptures in the female subgroup (*p* = 0.035, OR: 2.3 95% CI: 1.1–5.5). An inferred haplotype in the *TNC* gene was also associated with the risk of Achilles tendinopathy. These results provide a proof of concept for the use of a customised pipeline for the exploration of a larger genomic dataset. This approach, using previous research to guide a targeted analysis of the data has generated new genetic signatures in the biology of musculoskeletal soft tissue injuries.

## Introduction

Musculoskeletal soft tissue injury is an encompassing term used to describe pathologies affecting tendons, ligaments and skeletal muscles [1]. These phenotypes commonly present at clinical practices, with patients reporting impaired sporting performance and decreased functional capacity [1]. The underlying biological origin of these injuries is complex and still highly debated. However, several risk factors have been proposed, including a person’s genetic profile (reviewed by September et al. 2016 and Rahim et al. 2016) [2,3].

To date, several DNA sequence variants have been associated with susceptibility for overuse and acute injuries such as Achilles tendinopathy, tennis elbow, rotator cuff injuries and ACL ruptures [2–7]. It is interesting to note that although tendons and ligaments are anatomically distinct structures with different primary functions, they are similar in molecular composition and share several morphological features [8]. It is therefore not surprising that there are similarities (shared) and differences (unique) in the loci implicated in these clinically distinct musculoskeletal injuries [2].

These previous studies have employed the use of candidate gene, case-control genetic association studies to test the association of predominantly singleton variants with the risk of injury susceptibility. This approach is hypothesis driven, whereby the candidate genes and the investigated variants are selected based on known biological function and an *a priori* hypothesis that the gene product is involved in injury development [9]. Although this method has served as the basic investigative strategy for much of the research within the field, it has an important limitation. Our current knowledge of the biological mechanisms underlying musculoskeletal soft tissue injuries is limited. Consequently, only a select number of genes and variants have been investigated. Therefore, the ability to identify all risk conferring loci within the genome, contributing only modestly to injury susceptibility, is limited using this approach alone.

Few studies have adopted next generation technologies such as whole exome sequencing (WES) or genome wide association studies (GWAS) to define the susceptibility profile of musculoskeletal soft tissue injuries. One familial study including fraternal twins, both of whom had sustained ACL ruptures, identified 11 plausible risk-conferring variants that may contribute to ACL ruptures of a non-contact nature [10]. Baird et al. (2014) [11] and Baker et al. (2017) [12] identified alternate loci associated with the risk of cruciate ligament ruptures in the domestic dog. Furthermore, two single nucleotide polymorphisms were found to significantly associate with the risk of rotator cuff tears in a GWAS by Tashjian et al. (2017) [13]. Conversely, a GWAS conducted by Kim et al. (2017) [14] was unable to identify any risk conferring variants associated with either Achilles tendinopathy or ACL ruptures using a false discovery rate less than 5%. The slow uptake of high throughput technologies in the study of musculoskeletal soft tissue injuries may be explained by the expense associated with such an approach, from initial sample preparation through to the sequencing process and bioinformatic analysis of the generated data. This is particularly true in complex, multifactorial conditions in which large sample sizes are required in order to achieve sufficient power to detect statistically significant associations using traditional statistical methods [15]. A novel approach is therefore required to facilitate the interrogation of the genome and assist in refining the biological motifs underpinning musculoskeletal soft tissue injuries. Whole genome sequencing of well phenotyped participants provides the most comprehensive variant coverage, allowing for in-depth analyses of the genetic basis of disease [16]. However, the associated cost and complexity of data analysis has limited its accessibility. Since many disease-related variants have been identified in protein encoding regions [15,17] sequencing only the exome provides a cost effective alternative to whole genome sequencing.

Therefore, the primary aim of this study was to apply a whole exome sequencing approach using exemplar controls and cases, to further assist in identifying genetic markers predisposing to chronic Achilles tendinopathy. The secondary aim involved determining whether any variants found to be associated with chronic Achilles tendinopathy were similarly associated with the risk of acute ACL ruptures. This was based on the *a priori* hypothesis that functional variants within genes encoding components of the extracellular matrix (ECM) may similarly influence the integrity of both tendons and ligaments. Furthermore, previous research has implicated several genetic loci predisposing for chronic phenotypes in the risk profile of acute musculoskeletal soft tissue injuries (reviewed Rahim et al. 2016) [2]. Therefore, to address the aims of the study, a customised WES analysis pipeline was developed and variants within targeted genomic regions were prioritised for further interrogation in larger, independent injury groups using a case-control genetic association study approach.

## Material and methods

### Participants

Approval of this study was obtained from the Research Ethics Committee of the Faculty of Health Sciences within the University of Cape Town (HREC ref: 176/2015 and 224/2013). Existing databases of individuals previously recruited for ongoing research were used for this study. Two independent South African sample groups were selected for further downstream applications. The first, a South African chronic Achilles tendinopathy group, consisted of healthy control participants (CON, n = 165) and individuals with clinically diagnosed Achilles tendinopathy (TEN, n = 123). The second group included control participants (CON, n = 232) and individuals with surgically diagnosed anterior cruciate ligament ruptures (ACL, n = 234). Approximately 60% of participants with ACL ruptures reported a noncontact mechanism of injury and were additionally analysed as a separate subgroup (NON, n = 135). The participants in both groups were of self-reported European Caucasian ancestry. Experienced sports physicians or orthopaedic surgeons diagnosed all injuries. Achilles tendinopathy was confirmed using inclusion and exclusion criteria as previously described [18], whereas ACL ruptures were confirmed using magnetic resonance imaging (MRI), arthroscopy and during reconstructive surgery. Control participants were recruited from various sports clubs and gyms around Cape Town while the case participants were recruited from the Sports Science Orthopaedic Clinic at the Sports Science Institute of South Africa. All participants provided written informed consent in accordance with the Declaration of Helsinki, and completed a questionnaire regarding their personal particulars, physical activity and medical history. Approximately five mililitres of venous blood was previously obtained from each participant through the venipuncture of a forearm vein and collected in a vacuum ethylenediaminetetraacetic acid tube as previously described [19–21]. Blood samples were stored at -20°C until total DNA extraction was performed, using the protocol as described by Lahiri and Nurnberger (1991) [22] and later modified by Mokone *et al*. (2005) [19].

### Whole exome sequencing

Chronic Achilles tendinopathy was selected as the phenotypic model of injury for the initial exome sequencing project. The previously recruited Achilles tendinopathy group has been well characterised and was therefore considered the most appropriate phenotype for further interrogation into the genetic basis of disease [18,19]. From this original group, a subset of exemplar asymptomatic controls (n = 10, 6 males) and exemplar cases with chronic Achilles tendinopathy (n = 10, 6 males), representing divergent extremes of the phenotype spectrum were selected for WES. All cases reported the early onset of initial symptoms (35 years of age or younger), suffered chronic bilateral mid-portion Achilles tendinopathy and/or reported multiple Achilles tendon injuries (confirmed by a sports physician). The selected controls were unrelated, older than 47 years of age at time of recruitment, physically active and reported no previous tendon or ligament injuries. Genomic DNA extracted from each of these 20 participants was prepared according to the requirements of the WES service provider (Otogenetics, United States). All samples passed Otogenetics’ quality screening.

Individual samples were sequenced at paired ends on the Illumina HiSeq 2000/2500 platform at 30X coverage using the Agilent V5+UTR (71Mbp) capture kit (Agilent Technologies, Santa Clara, United States). The average read length on this platform is 106 base pairs. The Agilent V5+UTR capture kit targets all exonic and untranslated regions including miRBase miRNA genes. Sequencing read quality control was performed using FastQC (https://www.bioinformatics.babraham.ac.uk/projects/fastqc/). All samples passed pre- and post-alignment quality control with no trimming of reads required.

Forward and reverse sequencing reads for each sample were aligned to the hg19 (GRCh37) human genome build using Novoalign (Novocraft Technologies, Selangor, Malaysia). Samples which demonstrated insufficient coverage on the first sequencing run, were sequenced again on the same platform. For these samples, the sequencing alignment map (SAM) files were merged using Samtools ‘reheader’ and Picard ‘MergeSamFiles ‘ [23]. Samtools was used to convert SAM files to BAM files (binary version of SAM files) for condensed storage and further analysis. Duplicate reads were removed using Picard (https://broadinstitute.github.io/picard/). The Genome Analysis ToolKit (GATK) was then used for insertion-deletion realignment, base quality score recalibration and variant calling (https://software.broadinstitute.org/gatk/). Quality evaluation was performed using the GATK hard-filtering guidelines to produce a set of high-likelihood variants for each exome. The resulting variant call format (vcf) file was annotated using the web version of ANNOVAR (wANNOVAR) [24].

### Variant filtering

A total of 204 807 variants meeting the quality score threshold were called in the 20 samples included in this study. Variants where genotype calls were missing in two or more samples in either of the control or case groups were excluded due to sample size concerns (n = 1916). Variants called with more than two alleles (multi-allelic loci, n = 3 435) were extracted from the dataset as they did not form part of the focus of this initial study. These variants were stored for future analyses. Since tendinopathy is a common, multifactorial condition with no clear inheritance pattern, variants were not filtered on inheritance pattern. Allele frequencies were calculated in the sequenced control and case groups for each variant. An allele frequency difference of ≥30% was selected as a cut-off to identify variants of interest for further analysis. Hereafter, this frequency difference is referred to as the “allele frequency difference threshold”. This conservative threshold was selected based on the previous observation that an allele or genotype frequency difference of between 10–20% between control and case groups in genetic association studies usually indicated a significant association using traditional statistical methods. Furthermore, acknowledging the fact that (i) the biology of Achilles tendinopathy is multifactorial, (ii) a certain number of variants which are associated with predisposition may likely be common to both controls and cases and (iii) only a small number of samples were sequenced (n = 20), a 30% allele frequency difference was proposed as a conservative indicator of potential significance.

### Targeted gene analysis and variant prioritisation

To address the aims of this study, all variants identified by WES were filtered for those which met the allele frequency difference threshold. Given the limitations of the previous candidate variant approach, all variants located within genes implicated in genetic association studies were extracted and screened to identify potential risk modifying effects which were previously missed using the former approach. In addition, variants which mapped to any of the 35 genes identified as strong tendinopathy candidate genes in a previously published study using the BioOntological Relationship Graph (BORG) semantic database were also extracted for further interrogation [25].

**Fig 1:**
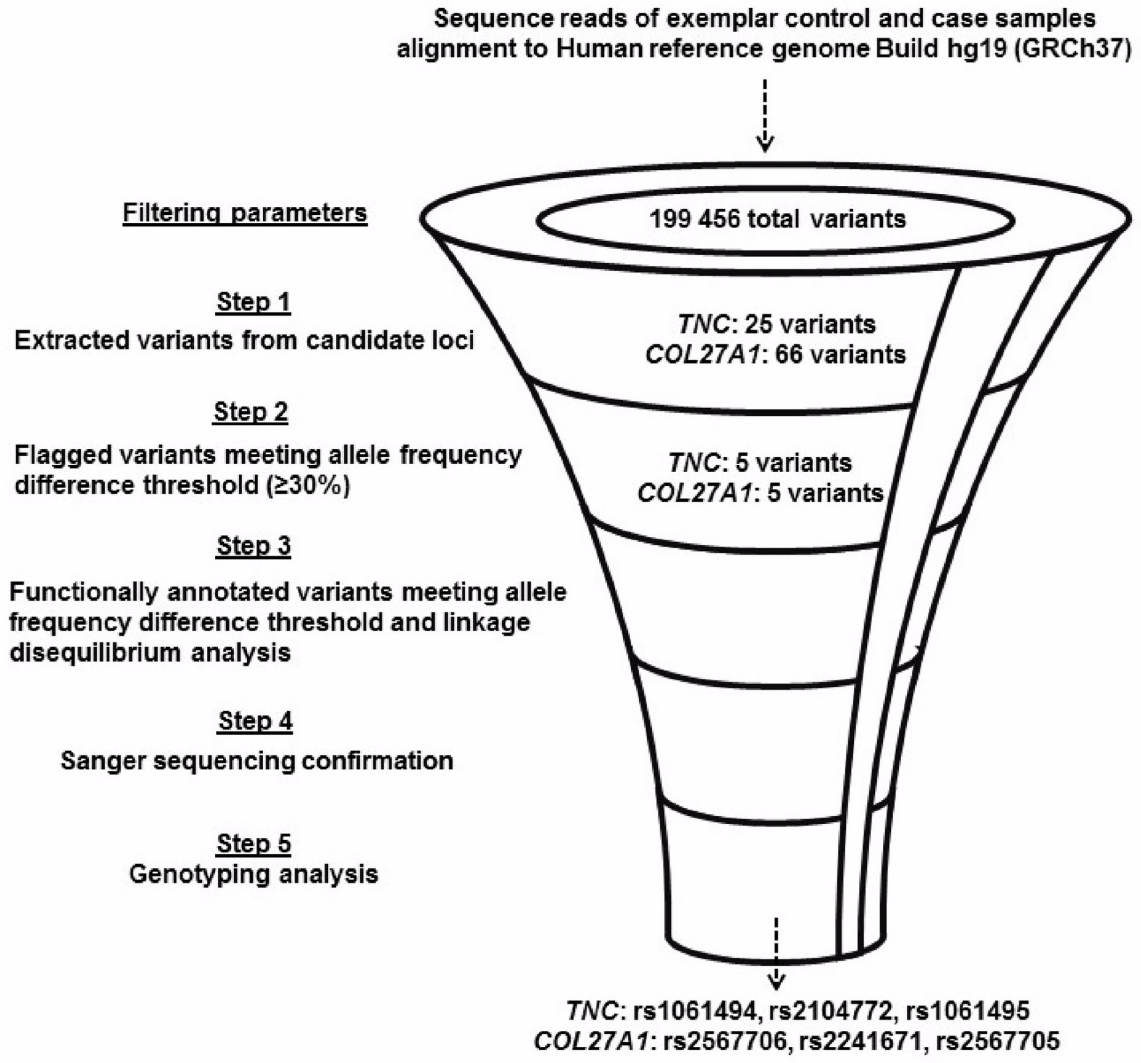
Customised filtering strategy used to prioritise variants identified by whole exome sequencing for interrogation in injury sample groups. Diagram describing the stepwise process of mining the data generated by whole exome sequencing using a customised tiered filtering strategy. An exemplar application of the filtering strategy is presented, whereby variants within the *TNC* and *COL27A1* genes were prioritised for genotyping. After alignment and post-alignment processing and the removal of variants with missing genotype data or multiple alleles, a total of 199 456 variants were identified across the 20 sequenced exomes. Step 1 involved extracting all variants identified by WES mapping to the candidate loci. Step 2 involved mining for variants with an allele frequency difference of ≥30% between the sequenced controls and cases. Step 3 involved the functional annotation of candidate variants using a several open, online bioinformatic resources. Linkage analysis using 1000 Genomes Project data (phase-3) was used to identify tagged loci from selected candidate variants. All variants prioritised for genotyping were confirmed using Sanger sequencing in step 4. Step 5 involved genotyping candidate variants in the larger injury sample groups.

To illustrate the proof of concept of using WES together with a targeted gene approach to define the predisposing genomic intervals for injury, the *COL27A1* (chr9q32) and *TNC* (chr9q33.1) genes which were previously associated with injury risk [18,26], were prioritised for further interrogation. All variants that localised to these genes and met the allele frequency difference threshold were extracted, mapped and functionally annotated using the National Center for Biotechnology Information (NCBI) database (https://www.ncbi.nlm.nih.gov/), and Ensembl (https://www.ensembl.org/index.html). Tagged SNPs were identified using the 1000 Genomes Project data (phase-3) [27] and the WES allele frequencies. To confirm pairwise linkage, putative tagged loci were genotyped in 10% of all samples in the Achilles tendinopathy group. All variants prioritised in the WES study were verified by Sanger sequencing (Inqaba Biotechnical Industries, Pretoria, South Africa) prior to genotyping. An illustrative overview of the customised filtering strategy is provided (Fig 1).

Variants were selected for genotyping in the larger Achilles tendinopathy and ACL rupture sample groups based on likely functional impact annotations, coding effects (splice, nonsense, missense variations), and linkage disequilibrium (Table 1). The three *TNC* variants prioritised for investigation were rs1061494 (T/C; exon 4), rs2104772 (A/T; exon 17) and rs1061495 (T/C; exon 18). The three *COL27A1* variants prioritised for investigation were rs2567706 (A/G; exon 3), rs2241671 (G/A; exon 3), and rs2567705 (A/T; exon 3). The rs1061495 (*TNC*) variant demonstrated an allele frequency difference of only 25% between the sequenced controls and cases. However, due to its location in relation to i) previously associated variants or ii) the newly identified WES candidates, in addition to its predicted coding effect, this variant was included in the analysis. Similarly, the *TNC* rs1138545 variant did not meet the allele frequency difference threshold but has previously been associated with degenerative rotator cuff tears and was therefore prioritised for investigation [5].

**Table 1:** The *TNC* and *COL27A1* variants prioritised for genotyping after exome sequencing and the application of a customised, tiered filtering strategy.

SNP ID	Alleles		MAF	Chromosome position	Variant location	Coding effect	AA change	Allele difference (%)
	Ref	Alt						
***TNC* variants**								
rs944510	C	T	0.44 (T)	9: 117853022	Exon 2	SYN	V-V	0.35
rs2992147	C	T	0.44 (C)	9: 117849314	Exon 3	SYN	V-V	0.35
rs1061494	C	T	0.44 (C)	9: 117846580	Exon 4	NON-SYN	Q-R	0.35
rs1138545	C	T	0.18 (T)	9: 117835899	Exon 10	NON-SYN	R-H	0.10
rs2104772	T	A	0.42 (A)	9: 117808785	Exon 17	NON-SYN	I-I	0.45
rs1061495	T	C	0.26 (C)	9: 117804544	Exon 18	SYN	T-T	0.25
***COL27A1* variants**								
rs2567706	G	A	0.27 (G)	9: 116930194	Exon 3	NON-SYN	Q-R	0.30
rs2241671	G	A	0.39 (A)	9: 116931099	Exon 3	NON-SYN	A-T	0.30
rs2808770	C	T	0.27 (C)	9: 116931445	Exon 3	NON-SYN	I-T	0.30
rs2567705	T	A	0.38 (T)	9: 116931666	Exon 3	NON-SYN	I-F	0.30
rs2808771	T	C	0.27 (T)	9: 116931737	Exon 3	SYN	G-G	0.30

*TNC*: Gene encoding the tenascin-C glycoprotein; *COL27A1*:Gene encoding α1 chain of type XXVII collagen. SNP ID: dbSNP accession number. MAF: Minor allele frequency according to European 1000 Genomes project data (phase-3), Chromosome Position: Chromosome location on the Human GRCh37.p13 Genome release. Coding effect: SYN (synonymous), NON-SYN (non-synonymous). AA change: Amino acid substitution at locus. The allele frequency difference between exemplar control and case groups reported in the WES data output as a percentage. V: Valine, Q: Glutamine, R: Arginine, H: Histidine, I: Isoleucine, L: Lysine, T: Threonine, A: Alanine, F: Phenylalanine, G: Glycine.

### Case-control genetic association analysis

The independent ACL sample group was introduced at this stage of the study, to assess whether the WES prioritised variants were associated with musculoskeletal soft tissue injuries other than that of Achilles tendinopathy. All case-control genetic association analyses were conducted in accordance with the strengthening the report of genetic association studies (STREGA) statement for reporting the results of genetic association studies [28]. The QUANTO v1.2.4 program was used to calculate the statistical power based on the respective sample sizes (http://biostats.usc.edu/software). All control and case participants were genotyped for the selected variants within the *TNC* and *COL27A1* genes using fluorescence-based Taqman single nucleotide polymorphism (SNP) genotyping assays (Applied Biosystems, Foster City, California, USA). The CON and TEN samples in the Achilles tendinopathy sample group were previously genotyped for the *TNC* rs2104772 variant and the results presented by Saunders et al. 2012 [26]. With permission, these genotypes, in addition to 30 extra Achilles tendinopathy cases and 34 controls genotyped in this current study, were included for haplotype analysis. For quality control purposes, positive and DNA-free control samples were included in each 96-well polymerase chain reaction (PCR) plate. Two independent investigators confirmed genotype calls and all laboratory work was conducted at the Division of Exercise Science & Sports Medicine within the University of Cape Town. It must be noted that results are presented for all variants in the male and female combined group, but only for those of significance in the independent sex groups in the ACL study group.

### Statistical analysis

Statistical analysis was conducted using the R programming environment (https://www.r-project.org/). Descriptive analyses of quantitative variables were represented as means and standard deviation (SD) while frequencies were determined for qualitative participant characteristics. A one-way analysis of variance (ANOVA) test was used to examine the characteristic differences amongst the sample groups and the possible genotype effects on age, height, weight, body mass index (BMI) and sex. The genetics and SNPassoc packages were used to estimate genotype and allele frequencies and exact Hardy-Weinberg equilibrium (HWE) probabilities for the controls and cases in the two independent groups [29,30]. Several non-genetic parameters were considered potential confounders and were corrected for in the logistic regression models testing for the association of genotypes and alleles with musculoskeletal soft tissue injuries. These confounding factors were equally weighted within the regression models and entered by means of a forward method. The haplo.stats package was used to test for the association between inferred haplotypes and the risk of injury [31]. The strength of association between the inferred haplotypes and the phenotype is reflected by the score statistic (Haplo.score). Negative values infer decreased risk, while positive scores infer increased risk. Only haplotypes with a total frequency >5% were presented. Statistical significance was accepted when *p*<0.05. No adjustments for multiple testing were made as it has been suggested that statistical corrections, including Bonferroni, overcorrect for an inflated false positive rate and significantly reduce the power in genetic association studies, in which linkage disequilibrium exists between loci [32].

## Results

The average depth of coverage for all samples was 49X with 90% of target bases sequenced at a coverage higher than 15X. A total of 199 456 variants meeting the quality score threshold were called in the 20 sequenced samples.

**Fig 2:**
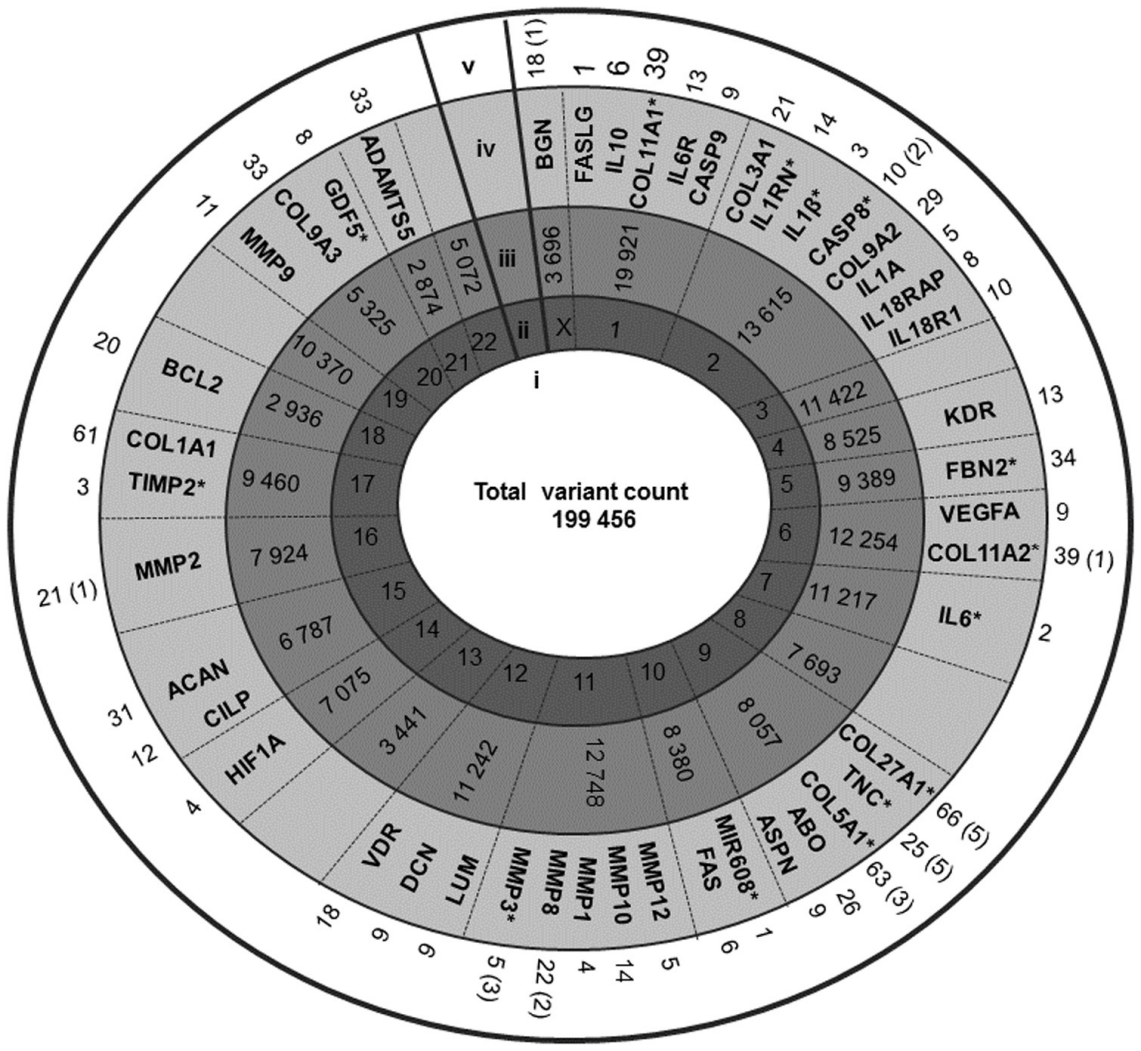
Distribution of variants identified by whole exome sequencing within genes previously associated with the risk of musculoskeletal soft tissue injuries. Schematic diagram demonstrating the number and distribution of variants identified through whole exome sequencing of ten exemplar controls and ten exemplar cases. (i) Total number of variants identified across the 20 sequenced exomes. (ii) The autosomes numbered from 1–22, and the X gonosome. The Y gonosome is not presented due to its size relative to the other chromosomes. A total of 32 variants were called on the Y chromosome. (iii) Total number of variants identified mapping to each chromosome using WES. (iv) Genes previously associated with risk of Achilles tendinopathy and other musculoskeletal soft tissue injuries mapping to the respective chromosomes. (*) Asterix indicates genes specifically associated with Achilles tendinopathy. (v) Total number of variants identified mapping to the respective genes with number of variants that meet the allele frequency difference threshold in parentheses.

**Fig 3:**
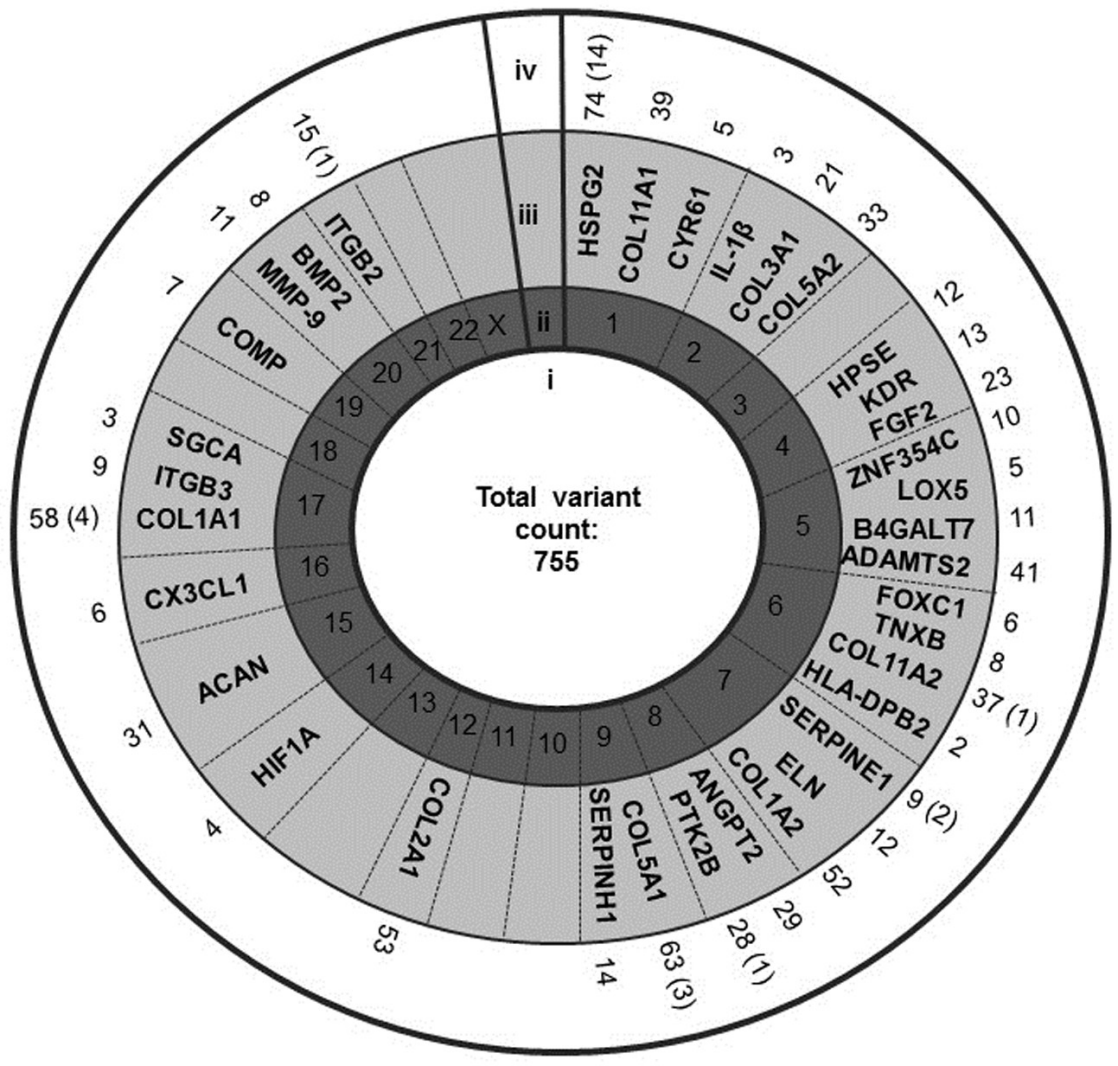
Distribution of variants identified by whole exome sequencing within genes proposed as plausible candidates for Achilles tendinopathy using the BioOntological Relationship Graph database. Schematic diagram demonstrating the number and distribution of variants identified by WES mapping to the 35 genes identified as plausible candidates for Achilles tendinopathy using BORG. (i) Total number of variants identified mapping to the 35 BORG candidate genes. (ii) The autosomes numbered from 1–22, and the X gonosome. (iii) Names of BORG identified candidate genes. (iv) Total number of variants identified mapping to BORG identified genes candidates with number of variants meeting the allele frequency difference threshold in parentheses.

A total of 4544 variants in 2127 genes met the allele frequency difference threshold. With respect to the genes previously associated with Achilles tendinopathy using a candidate gene approach, 338 variants were identified, 19 demonstrating allele frequency differences equal to or above the 30% threshold (Fig 2). The rs2104772 (A/T) variant, within the previously associated *TNC* gene [26], demonstrated the highest allele frequency difference between the sequenced controls and cases (45%). Furthermore, 757 variants, 26 of which met the allele frequency difference threshold, mapped to the 35 genes previously proposed as plausible candidate genes for tendinopathy risk using the BORG *in silico* approach (Fig 3) [25]. The heparan sulfate proteoglycan 2 (*HSPG2)* gene on chromosome 1 was the locus with the greatest number of variants (n = 74), 14 of which met the allele frequency difference threshold.

Three *TNC* variants identified by WES and meeting the allele frequency difference threshold were in complete linkage disequilibrium with one another. Specifically, the T allele of rs1061494 (C/T) corresponded to the T allele of the rs2992147 (T/C) and the C allele of rs944510 (C/T). The D’ measure and coefficient of correlation (r^2^) was 1.000 between all three variants respectively [27]. The remaining WES prioritised variants were not tagged loci and for that reason were genotyped in the larger sample groups. The participant characteristics of the Achilles tendinopathy and ACL groups were previously described [18,21,33]. No genotype effects on participant characteristics were noted for the investigated *TNC* variants (S1 Table). The genotype and minor allele frequency distributions of the *TNC* variants in the control and case samples for the two independent sample groups, together with the test scores for HWE are additionally provided (S2 and S3 Tables).

**Fig 4:**
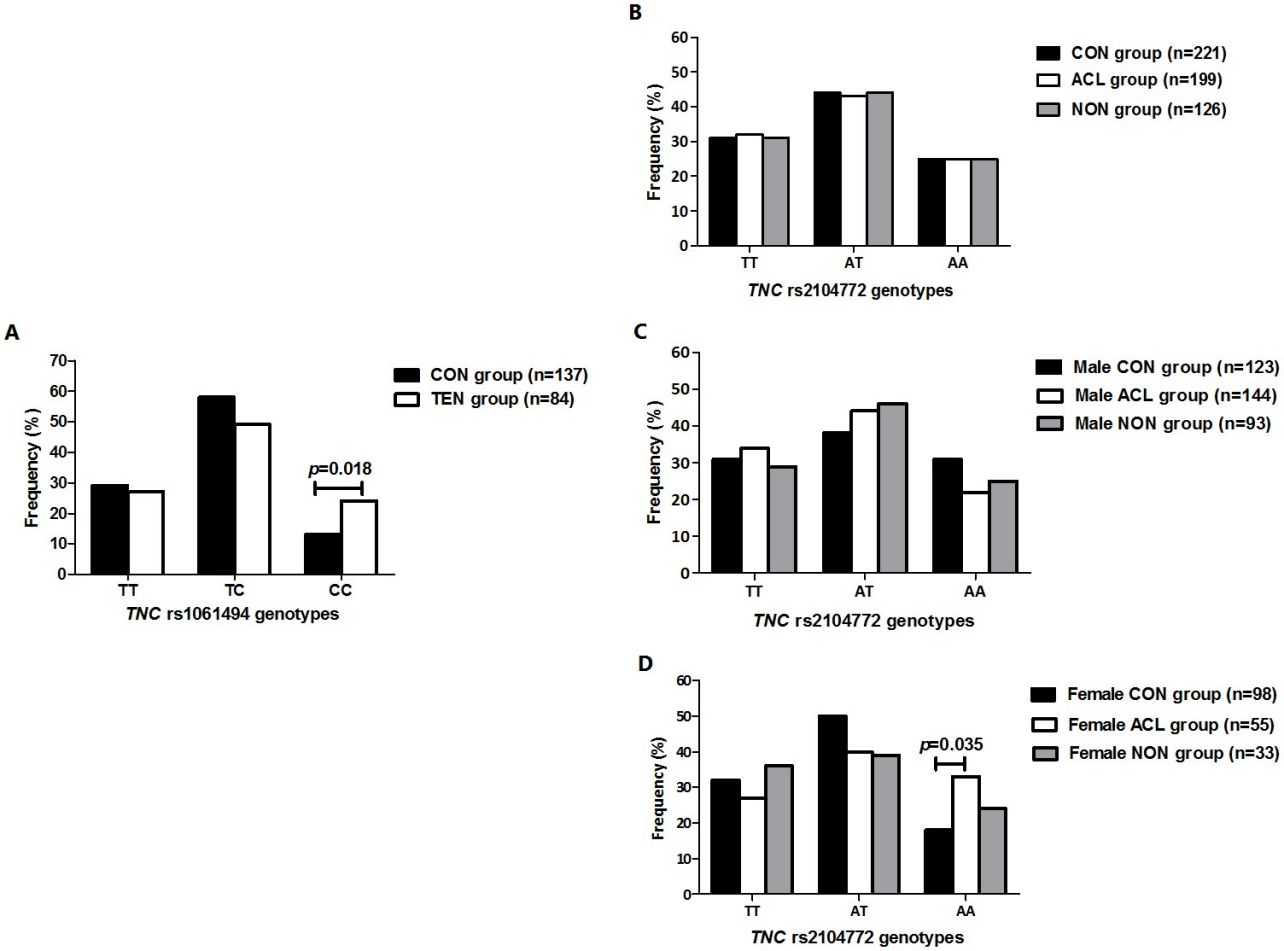
Genotype frequency differences of the *TNC* rs1061494 and rs2104772 variants in the Achilles tendinopathy and ACL rupture groups. Genotype frequency distribution of the *TNC* rs1061494 (C/T) variant in the (A) Achilles tendinopathy sample group. Genotype frequency distributions of the *TNC* rs2104772 (A/T) variant in the (B) ACL sample group, (C) male ACL subgroup and (D) female ACL subgroup. CON: Control participants; TEN: Cases with Achilles tendinopathy; ACL: Cases with ACL ruptures; NON: Subgroup of cases reporting a non-contact mechanism of ACL injury. Statistically significant differences in genotype frequency between the groups are depicted on the graphs, with *p*-values adjusted for confounders including age and sex in the Achilles tendinopathy sample group and age, sex and weight in the ACL rupture sample group. The number of participants in each group (n) is in parentheses.

No significant differences in the genotype and allele frequency distributions were noted between the CON and TEN groups in the Achilles tendinopathy sample group for two of the three genotyped variants; rs1138545 (C/T) CON vs. TEN (*p* = 0.437); rs1061495 (T/C) CON vs. TEN (*p* = 1.000) (S2 Table). A significant difference was however noted for rs1061494 (T/C) between the control and case groups (*p* = 0.049). Specifically, the CC genotype was significantly underrepresented in the CON group compared to the TEN group (CON: 13%, n = 18, TEN: 24%, n = 20; *p* = 0.018, OR: 2.5 95%, CI: 1.2–5.1) (Fig 4A).

**Fig 5:**
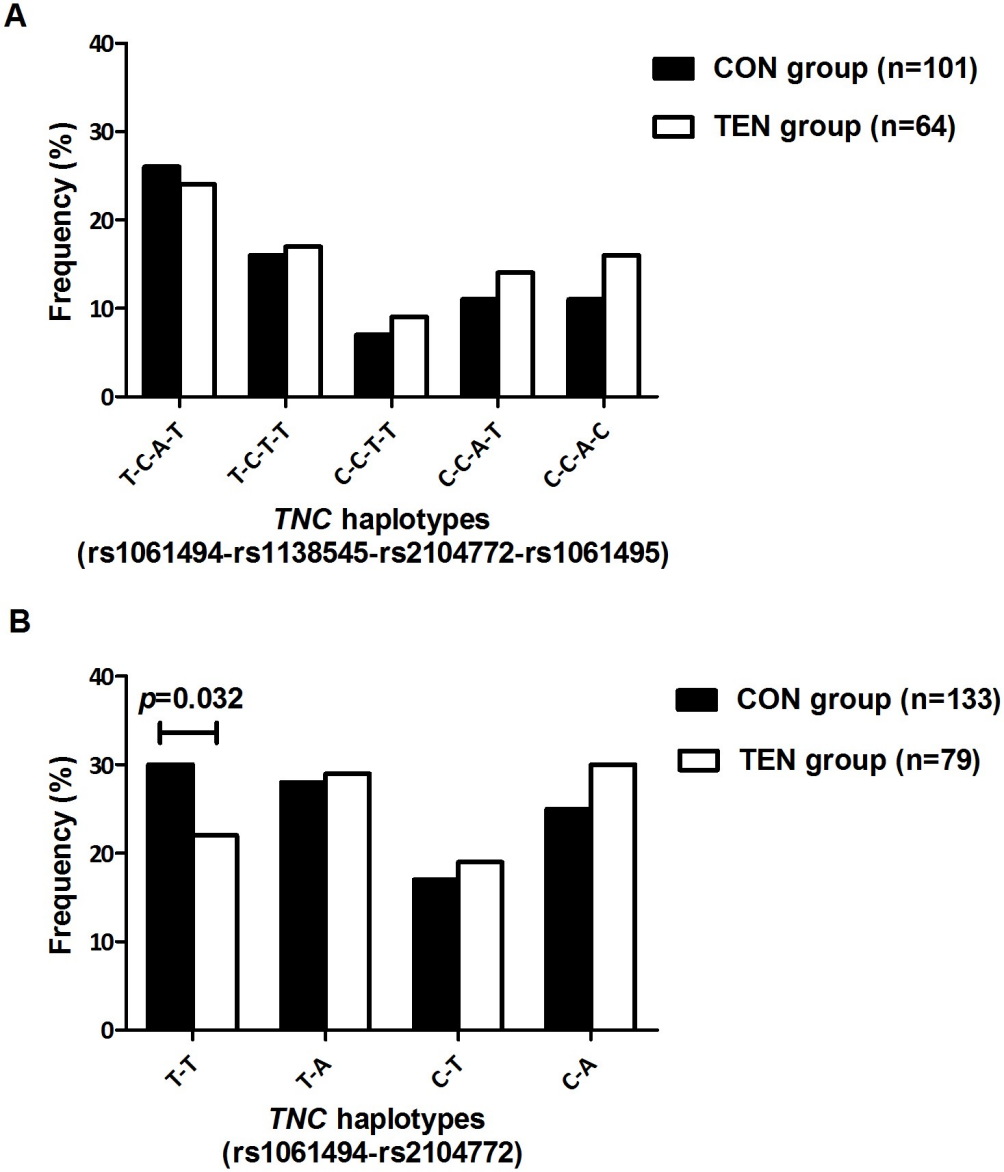
Frequency distribution of *TNC* haplotypes in the Achilles tendinopathy sample group. (A) Frequency distribution of the inferred haplotypes constructed from the *TNC* rs1061494, rs1138545, rs2104772 and rs1061495 variants in the Achilles tendinopathy sample group. (B) Frequency distribution of the inferred haplotypes constructed from the *TNC* rs1061494 and rs2104772 variants in the Achilles tendinopathy sample group. CON: Control participants; TEN: Achilles tendinopathy cases. Statistically significant differences in haplotype frequency between the groups are depicted on the graph, with *p*-values adjusted for age and sex. The number of participants (n) in each group is in parentheses.

No significant differences in the genotype and allele frequency distributions were noted between the CON group, ACL group and NON subgroup in the ACL injury sample group for the genotyped variants; rs1061494 (T/C) CON vs. ACL (*p* = 0.917), CON vs. NON (*p* = 0.598); rs1138545 (C/T) CON vs. ACL (*p* = 0.489), CON vs. NON (*p* = 0.356); rs2104772 (T/A) CON vs. ACL (*p* = 0.846), CON vs. NON (*p* = 0.816) and rs1061495 (T/C) CON vs. ACL (*p* = 0.169), CON vs. NON (*p* = 0.129), with the exception of the rs2104772 variant. The genotype frequency distribution of rs2104772 (T/A) was significantly different between controls and cases when only female participants were evaluated in the ACL sample group (*p* = 0.048), with the AA genotype significantly underrepresented in the CON group (18%, n = 18) compared to the group of cases (33%, n = 18, *p* = 0.035; OR: 2.3 95% CI: 1.1–5.5) (Fig 4D). Deviations from HWE have been noted (S2 and S3 Tables). Haplotypes were inferred using the genotype data from the four investigated *TNC* variants, rs1061494 (T/C), rs1138545 (C/T), rs2104772 (T/A) and rs1061495 (T/C). No significant differences in the frequency distributions of the inferred haplotypes were noted between the CON and TEN participants in the Achilles tendinopathy sample group (Fig 5A). However, a significant frequency difference was noted for the *TNC* T-T inferred haplotype, constructed from the rs1061494-rs2104772 variants in the Achilles tendinopathy sample group. Specifically, the frequency distribution of the T-T inferred haplotype was significantly overrepresented in the CON group (30%, n = 40) compared to the TEN group (22%, n = 17) (Dominant model: *p* = 0.032, Haplo.score: -2.146) (Fig 5B).

No significant differences in the frequency distribution of the inferred haplotypes constructed from the four investigated *TNC* variants were noted between the CON and ACL groups and similarly between the CON group and NON subgroup when all participants were analysed (*p* = 0.747, *p* = 0.600) (Figure A in S1 File). Similarly, the frequency distributions of the inferred haplotypes constructed from the two variants associated with the risk of Achilles tendinopathy (rs1061494-rs2104772) were not significantly different between the CON and ACL groups (*p* = 0.945) and CON group and NON subgroup (*p* = 0.908) (Figure B in S1 File). Furthermore, no significant differences in the distribution of the inferred haplotypes constructed from the four investigated variants were noted when male CON participants were compared to male ACL and male NON participants (*p* = 0.735, *p* = 0.703) (Figure C in S1 File). The frequency of the haplotypes constructed from the two variants associated with the risk of AT (rs1061494-rs2104772) were also similarly distributed between the CON and ACL group (*p* = 0.730) and CON and NON subgroup (*p* = 0.836) when male participant were analysed independently (Figure D in S1 File).

Four of the WES identified *COL27A1* variants meeting the allele frequency threshold were in high linkage disequilibrium. Specifically, the A allele of rs2567706 (A/G) corresponded to the G of rs2567707 (G/A), the T allele of rs2808770 (T/C) and the C allele of rs2808771 (C/T) respectively. The D’ measures were reported at 1.000 and the coefficient of correlation ranged between 0.900–1.000 for all pairwise comparisons [27]. The remaining WES identified variants mapping to *COL27A1* were in linkage equilibrium and for that reason were genotyped in the larger sample groups. No genotype effects on participant characteristics were noted for the investigated variants (S4 Table).

No significant differences in the genotype and allele frequency distributions were noted between the CON and TEN participants in the Achilles tendinopathy sample group for all three genotyped variants; rs2567706 (A/G) CON vs. TEN (*p* = 0.778); rs2241671 (G/A) CON vs. TEN (*p* = 0.964); rs2567705 (A/T) CON vs. TEN (*p* = 0.626) (S5 Table). Similarly, no significant differences in the genotype and allele frequency distributions were noted between the CON, ACL and NON participants in the ACL sample group for all three *COL27A1* variants; rs2567706 (A/G) CON vs. ACL (*p* = 0.623), CON vs. NON (*p* = 0.822); rs2241671 (G/A) CON vs. ACL (*p* = 0.787), CON vs. NON (*p* = 0.751); rs2567705 (A/T) CON vs. ACL (*p* = 0.762), CON vs. NON (*p* = 0.504) (S6 Table).

Haplotypes were inferred using the genotype data from the three investigated *COL27A1* variants, rs2567706 (A/G), rs2241671 (G/A) and rs2567705 (A/T). No significant differences in the distribution of the inferred haplotypes constructed from all *COL27A1* variants were noted between the control and case participants in the Achilles tendinopathy *(p* = 0.636) (Figure A in S2 File) and ACL rupture sample groups (CON vs. ACL: *p* = 0.234, CON vs. NON: *p* = 0.089) (Figure B in S2 File). Similar results were observed when male participants in the ACL rupture sample group were evaluated independently (CON vs. ACL: *p* = 0.211, CON vs. NON: *p* = 0.845) (Figure C in S2 File).

## Discussion

Currently, our understanding of the biological mechanisms underpinning musculoskeletal soft tissue injuries is limited. Consequently, only a select number of genes have been prioritised for exploration and only variants presented in the literature as being associated with biological processes proposed to be involved in musculoskeletal soft tissue injuries, have been investigated. To date, no more than 80 variants have been profiled in musculoskeletal disorder association studies, which have had varying levels of replication success [2]. These studies have predominantly used an *a priori* hypothesis driven, candidate variant approach. However, it is unlikely that all risk modifying loci, predisposing for these complex, multifactorial conditions will be identified using this method alone. It is for this reason, that this study adopted a multifaceted approach, whereby previous experimental research was used to guide a targeted analysis of the WES data. Genes previously implicated in injury susceptibility were prioritised during the initial data mining steps and novel candidate variants were prioritised for analysis based on allele frequency differences in the WES data between sequenced controls and cases, putative functional and coding effects and population frequencies.

The exome sequencing of 20 exemplar controls and cases identified 199 456 high quality variants, 4544 of which demonstrated an allele frequency difference of ≥ 30% between the sequenced controls and cases. Interestingly, only 19 (<1%) variants meeting the allele frequency threshold mapped to the genes previously implicated in the aetiology of Achilles tendinopathy. The majority of these variants localised to the neighbouring *TNC* and *COL27A1* genes and were prioritised for further interrogation in this study.

The *TNC* gene encodes a hexameric extracellular matrix glycoprotein that is highly expressed during embryonic development and in tissues subjected to tensile loading, including tendons and ligaments. Moreover, transient expression is observed during wound healing, tissue remodelling and more persistently in pathological conditions [34–37]. Analysis of the *TNC* WES data highlighted three tagged loci including, rs944510 (C/T), rs2992147 (T/C) and rs1061494 (T/C) respectively. The missense rs1061494 variant served as a proxy for the other tagged loci. The CC genotype of the rs1061494 variant, which is located within the first constitutively expressed fibronectin type III (FNIII) repeat domain of the peptide, inferred a 2.5-fold increased risk for chronic Achilles tendinopathy.

Recently, variants within the *TNC* gene have been acknowledged as expression quantitative trait loci (eQTL) [38,39]. By definition, these are common variants in the genome that determine phenotypic traits including, alterations in transcript levels (eQTL) and protein abundance (pQTL) [40,41]. Interestingly, rs1061494 is a predicted *cis*-eQTL in testis tissue, whereby the CC genotype is predicted to associate with increased expression [42]. In the current study, the CC genotype was overrepresented in the cases with Achilles tendinopathy, suggesting potential increased *TNC* expression in the affected group. However, whether these transcript-altering properties of rs1061494 in testis tissue translate to expression alterations in dense connective tissue, requires experimental validation.

Another noteworthy finding pertained to the *HSPG2* gene, encoding the perlecan modular proteoglycan [43]. This gene demonstrated the greatest density of variants meeting the allele frequency difference threshold. Interestingly, perlecan is a known biological substrate of tenascin-C, with binding sites in the FNIII repeat domains [34]. Therefore, it can be hypothesised that the concentration of variants within binding site sequences may alter the ability of these two proteins to bind, thereby impacting matrix regulation and modulating susceptibility to injury.

**Fig 6:**
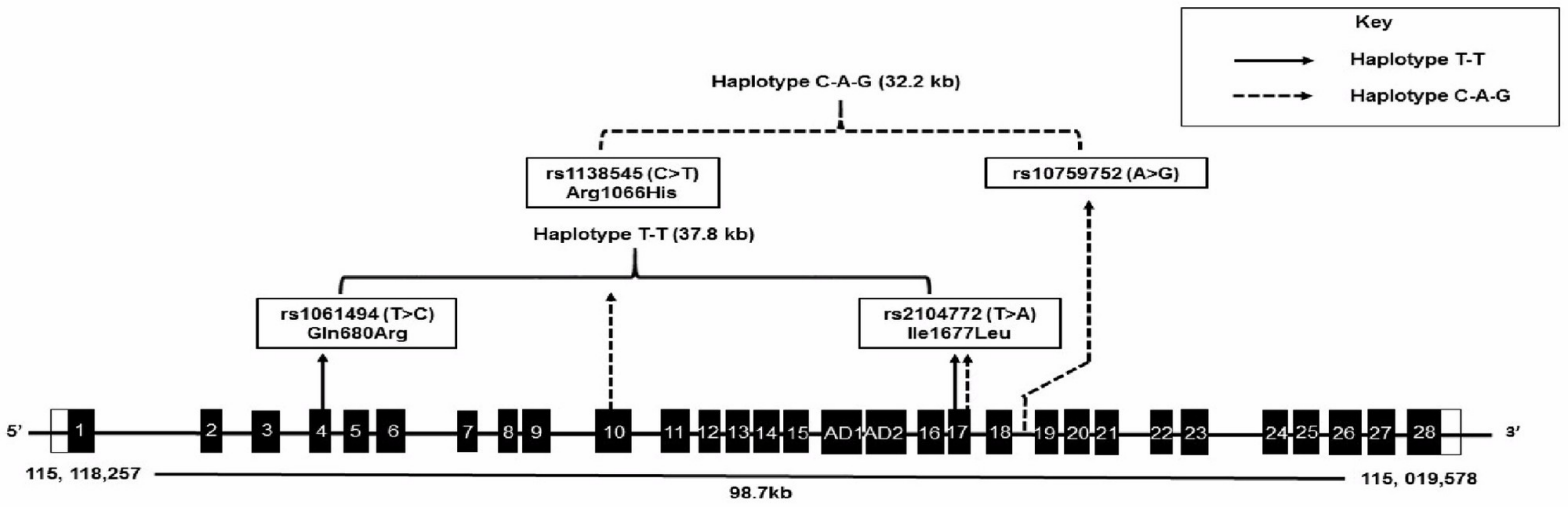
Risk-associated inferred haplotypes mapping to the *TNC* genomic interval. Diagram demonstrates two overlapping *TNC* inferred haplotypes associated with the risk of musculoskeletal soft tissue injuries including Achilles tendinopathy (rs1061494-rs2104772) and recurrent rotator cuff injuries (rs1138545-rs2104772-rs10759752) [6]. The genomic boundaries of *TNC* are provided according to the Human GRCh38.p10 genome release. Translation of mRNA starts in exon 2 demonstrated by double dash. Although the genomic coordinates are provided in the 5’ to 3’ direction, the *TNC* gene is orientated on the antisense strand. Black bars: Exons. Horizontal lines: Introns. Diagram not drawn to scale.

In addition, this study identified a risk-associated haplotype spanning 37.8kb (chronic Achilles tendinopathy) across the *TNC* gene (Fig 6). Specifically, the inferred *TNC* T-T haplotype (rs1061494-rs2104772) was associated with decreased risk for chronic Achilles tendinopathy. Both variants have independently been associated with risk for Achilles tendinopathy, rs1061494 in the current study and rs2104772 in a previous study by Saunders et al. (2012) [26]. Moreover, rs2104772 maps within 2kb of a guanine-thymine dinucleotide repeat, the first variant proposed as a risk modifier for Achilles tendon injuries [18].

Interestingly, a novel three variant *TNC* inferred haplotype, C-A-G (rs1138545-rs2104772-rs10759752), was recently associated with the risk of large recurrent defects after rotator cuff surgery (Fig 6) [6]. The missense rs1138545 variant, described by the authors as the most influential marker of those associated, is located one amino acid away from the bridging sequence between the FNIII (5) repeat and FNIII (1A) repeat domains [5]. Furthermore, rs1138545 is in complete LD with rs7021589, a predicted *cis*-pQTL (protein quantitative trait loci) with significant regulatory effects on protein plasma levels [39]. These results reiterate locus heterogeneity among clinically distinct musculoskeletal soft tissue injuries, whereby some modifiers may be common to the predisposing genetic signatures, while other motifs are unique.

Taken collectively, the proposed biological functions of these variants, together with the data from the current and previous associations studies, provides support for the potential significance of this genomic interval in the profile predisposing for injury [5,6,26]. It must be noted that a deviation from HWE was observed for the rs1061494 and rs2104772 variants in the respective control groups. This may be due to the highly selective inclusion criteria of the control participants, which results in a group that is not representative of the general population but represents a subset of the population at a divergent end of the injury phenotype spectrum.

Approximately 800kb upstream from the associated *TNC* haplotype is the *COL27A1* gene. *COL27A1* has previously been associated with the risk of Achilles tendinopathy, specifically the rs946053 (T/G) variant in combination with other *TNC* variants [26]. The *COL27A1* gene encodes the α1 chain of type XXVII collagen, an atypical member of the fibrillar collagen family. The function of this protein family involves providing structural support and tensile strength to extracellular matrices, with *COL27A1* expression predominantly localised to cartilaginous structures [44]. Analysis of the WES data identified four non-synonymous variants in high linkage disequilibrium within *COL27A1* namely rs2567707 (G/A), rs2567706 (A/G), rs2808770 (T/C) and rs2808771 (C/T). The rs2567706 variant demonstrated the strongest pairwise linkage and for this reason, served as a proxy for the other tagged loci. However, in addition to rs2567706, no *COL27A1* variants were significantly associated with the risk of chronic Achilles tendinopathy or acute ACL ruptures.

According to the Sorting Intolerant from Tolerant (SIFT) [45] and Polymorphism phenotyping (Polyphen-2 v2) [46] online tools, none of the investigated variants were predicted to be damaging in effect (Table 2). Conversely, according to the Combined Annotation Dependent Depletion (CADD) framework for estimating the pathogenicity of genetic variation, the *TNC* rs2104772 and rs1138545 variants in addition to the *COL27A1* rs2567705 variant demonstrated CADD_phred scores >13, equivalent to a 95% probability that the variant has an effect functionally [47]. Similarly, the Fathmm_MKL tool for predicting the functional consequence of coding and non-coding single nucleotide variants, predicted the rs2104772 variant to be *‘deleterious’* [48].

**Table 2:** Predicted functional effects of the *TNC* and *COL27A1* variants prioritised for investigation after whole exome sequencing.

SNP ID	Coding effect	Functional effect predicted by				
		SIFT	PolyPhen-2	CADD	DANN	Fathmm_MKL
***TNC* variants**						
rs1061494	NON-SYN	T	B	0.62	0.54	N
rs1138545	NON-SYN	T	B	**17.19**	0.85	N
rs2104772	NON-SYN	T	B	**17.21**	0.70	**D**
rs1061495	SYN	-	-	-	-	-
***COL27A1* variants**						
rs2567706	NON-SYN	T	B	2.00	0.81	N
rs2241671	NON-SYN	T	B	0.00	0.36	N
rs2567705	NON-SYN	T	B	**16.51**	0.91	N

SNP ID: dbSNP accession number. Coding effect: NON-SYN (non-synonymous); SYN (synonymous). T: Tolerated; B: Benign; D: Deleterious; N: Neutral. Bold typeset indicates an estimate predicting for functional significance.

These results suggest that although not disease causing, the effects of these variants may be impressionable at the functional level. Taking this into consideration, together with the high prevalence of musculoskeletal soft tissue injuries, it is reasonable to hypothesise that these injuries are underpinned by a myriad of common, non-lethal variants, each of which contribute modestly to the molecular signature predisposing for injury. Another point to consider is that although this study supports the involvement of genetics in the biology of musculoskeletal soft tissue injuries, the intrinsic mechanisms resulting in injury are still not fully understood. Therefore, the level to which these loci contribute to a connective tissue’s ability to respond to load or its ability to adapt and resolve after repetitive stressors is still unknown. Therefore, in addition to prospective longitudinal risk factor analyses, the functional impact of these genetic variants within the cellular environment also needs to be explored.

Furthermore, it is evident from the number of variants identified through WES in relation to the number mapping to previously implicated genes, that much of the genome remains uncharacterised in the aetiology of common sporting injuries. In saying this, it is likely that many genes within the genome do not contribute to the aetiology of musculoskeletal injuries and therefore, do not confer risk. However, it is also plausible to assume that variants, currently of unknown location and function, possess important risk modifying capabilities. Therefore, these results support the need for further interrogation of the genome using multi-integrated approaches.

Exome sequencing has an important limitation in that it overlooks the potential effects of variants in intronic and regulatory regions of the gene. Furthermore, although this study made use of highly selected exemplar controls and cases, the small number of sequenced individuals means that important risk modifiers may have gone undetected. The size of the Achilles tendinopathy sample group also presented as a limitation, in addition to that of the female ACL subgroup. It is widely accepted that females are more predisposed to ACL ruptures, therefore it would be of interest to focus recruitment efforts on obtaining more female participants for future investigations. Future studies should aim to replicate these findings in other larger, independent populations in addition to the samples collected through the Genomics of injuries (GOINg) Consortium [49]. It is imperative that we facilitate in-depth explorations of the genome to identify risk-conferring variants, a process that is more likely to be accomplished through combining expertise and resources.

## Conclusions

These results provided proof of concept for the use of a customised analysis pipeline, together with a tiered filtering strategy, to explore a larger genomic dataset generated by WES. Due to financial constraints preventing the high throughput sequencing of a large sample of individuals, selection of exemplar controls and cases was an important project objective. Although the initial step of variant prioritisation was not based on statistical methods but rather a predetermined allele frequency difference threshold, the potential biological significance of the candidate variants was an important consideration. Using this approach, this study has identified a potential risk-modifying motif within the *TNC* gene for Achilles tendinopathy. Importantly, a change in the pattern of tenascin-C expression is documented in pathological tendons, a particularly significant observation owing to its strong ability to modulate cell behaviour.

Therefore, knowledge of the underpinning genetic signatures may assist in defining the multifactorial profiles predisposing for musculoskeletal soft tissue injuries. Although large prospective follow-up studies will be required to evaluate the efficacy of genetic screens to accurately predict injury risk, it is plausible to assume that this information may eventually be used by clinicians to develop personalised injury reduction and prehabilitation programmes targeted at strengthening tendons and ligaments.

## Supporting information

S1 File Frequency distribution of *TNC* haplotypes in the ACL rupture sample group. (A) Frequency distribution of the inferred haplotypes constructed from the *TNC* rs1061494, rs1138545, rs2104772 and rs1061495 variants in the ACL rupture sample group. (B) Frequency distribution of the inferred haplotypes constructed from the *TNC* rs1061494 and rs2104772 variants in the ACL rupture sample group. (C) Frequency distribution of the inferred haplotypes constructed from the *TNC* rs1061494, rs1138545, rs2104772 and rs1061495 variants in the male ACL rupture subgroup. (D) Frequency distribution of the inferred haplotypes constructed from the *TNC* rs1061494 and rs2104772 variants in the male ACL rupture subgroup. CON: Control participants; ACL: Cases with ACL ruptures; NON: Cases reporting a non-contact mechanism of injury. Statistically significant differences in haplotype frequency between the groups are depicted on the graph, with *p*-values adjusted for age and weight and sex when all participants were evaluated. The number of participants (n) in each group is in parentheses.
(TIF)

S2 File Frequency distribution of *COL27A1* haplotypes in the Achilles tendinopathy and ACL rupture sample groups. Frequency distribution of the inferred haplotypes constructed from the *COL27A1* rs2567706, rs2241671 and rs2567705 variants in the (A) Achilles tendinopathy and (B) ACL rupture sample groups, in addition to the (C) male ACL rupture subgroup. CON: Control participants; ACL: Cases with ACL ruptures; NON: Cases reporting a non-contact mechanism of injury. Statistically significant differences in haplotype frequency between the groups are depicted on the graph, with *p*-values adjusted for age and sex in the Achilles tendinopathy sample group and age, sex and weight in the ACL rupture sample group. The number of participants (n) in each group is in parentheses.
(TIF)

S1 Table Genotype effects of *TNC* variants, rs1061494, rs11384545, rs2104772 and rs1061495 on the physiological characteristics of participants. Values expressed as *p*-values. Effects on weight and BMI are adjusted for age and sex. Effects on height are adjusted for sex. *P*-values in bold typeset indicate significance (*p*<0.05). TEN: Achilles tendinopathy sample group. ACL: Anterior cruciate ligament rupture sample group.
(DOCX)

S2 Table Genotype, minor allele frequencies and HWE probabilities for the controls (CON) and cases with Achilles tendinopathy (TEN) in the Achilles tendinopathy sample group for the four *TNC* variants; rs1061494, rs1138545, rs2104772 and rs1061495 respectively. *P*-values are for difference in genotypes and minor allele frequencies between diagnostic groups adjusted for age at the time of recruitment for controls and at the time of injury for cases, in addition to sex. Bold typeset indicates significance (*p*<0.05). Participant count for genotypes presented in parenthesis and total count in italics. ^a^ rs1138545 CC genotype vs. CT + TT genotype. ^b^ rs1061495 TT genotype vs. CT + CC genotype.
(DOCX)

S3 Table Genotype, minor allele frequencies and HWE probabilities for the controls (CON) and cases with anterior cruciate ligament ruptures (ACL), including participants reporting a noncontact mechanism of injury (NON) in the ACL sample group for the four investigated *TNC* variants; rs1061494, rs1138545, rs2104772 and rs1061495 respectively. *P*-values are for difference in genotypes and minor allele frequencies between diagnostic groups adjusted for age and weight at the time of recruitment for controls and the time of injury for cases, in addition to sex. Bold typeset indicates significance (*p*<0.05). Participant count for genotypes presented in parenthesis and total count in italics. ^a^ rs1138545 CC genotype vs. CT + TT genotype. ^b^ rs1061495 TT genotype vs. CT + CC genotype.
(DOCX)

S4 Table Genotype effects of *COL27A1* variants, rs2567706, rs2241671 and rs2567705 on the physiological characteristics of participants. Values expressed as *p*-values. Effects on weight and BMI are adjusted for age and sex. Effects on height are adjusted for sex. P-values in bold typeset indicate significance (*p*<0.05). TEN: Achilles tendinopathy sample group. ACL: Anterior cruciate ligament rupture sample group.
(DOCX)

S5 Table Genotype, minor allele frequencies and HWE probabilities for the controls (CON) and cases with Achilles tendinopathy (TEN) in the Achilles tendinopathy sample group for the three *COL27A1* variants; rs2567706, rs2241671 and rs2567705 respectively. *P*-values are for difference in genotypes and minor allele frequencies between diagnostic groups adjusted for age at the time of recruitment for controls and at the time of injury for cases, in addition to sex. Bold typeset indicates significance *(p*<0.05). Participant count for genotypes presented in parenthesis and total count in italics. ^a^ rs2567706 AA genotype vs. AG + GG genotype.
(DOCX)

S6 Table Genotype, minor allele frequencies and HWE probabilities for the controls (CON) and cases with anterior cruciate ligament ruptures (ACL), including participants reporting a noncontact mechanism of injury (NON) in the ACL sample group for the three investigated *COL27A1* variants; rs2567706, rs2241671 and rs2567705 respectively. *P*-values are for difference in genotypes and minor allele frequencies between diagnostic groups adjusted for age and weight at the time of recruitment for controls and at the time of injury for cases, in addition to sex. Bold typeset indicates significance (*p*<0.05). Participant count for genotypes presented in parenthesis and total count in italics. ^a^ rs2567706 AA genotype vs. AG + GG genotype.
(DOCX)
