# Supplementary figures and images for: Defining the molecular signatures of Achilles tendinopathy and anterior cruciate ligament ruptures: A whole-exome sequencing approach
Source: PLoS One. 2018 Oct 25;13(10):e0205860. doi: 10.1371/journal.pone.0205860 (PMC6201890; doi:10.1371/journal.pone.0205860)

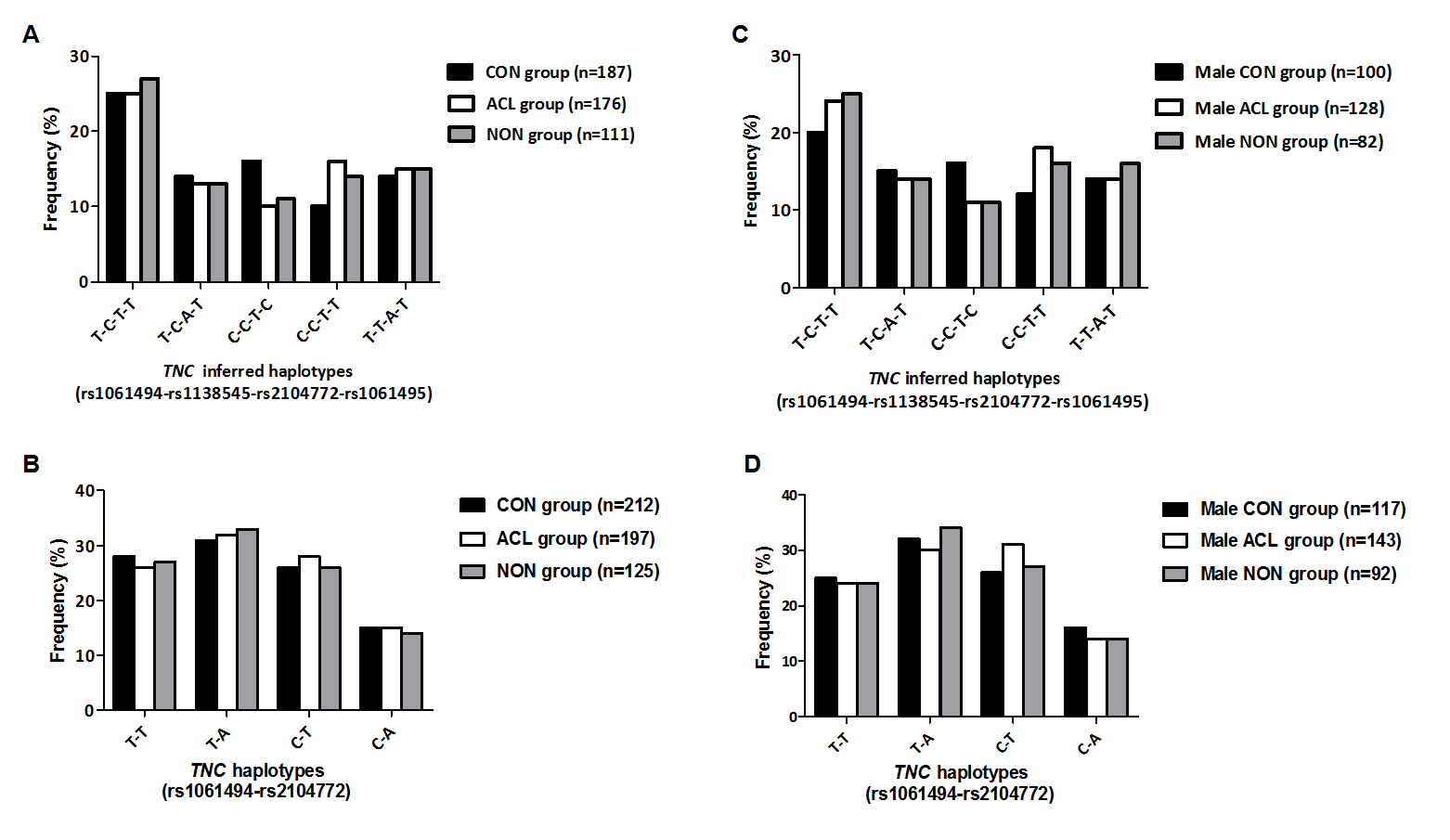

Supplement: S1 File — (A) Frequency distribution of the inferred haplotypes constructed from the TNC rs1061494, rs1138545, rs2104772 and rs1061495 variants in the ACL rupture sample group. (B) Frequency distribution of the inferred haplotypes constructed from the TNC rs1061494 and rs2104772 variants in the ACL rupture sample group. (C) Frequency distribution of the inferred haplotypes constructed from the TNC rs1061494, rs1138545, rs2104772 and rs1061495 variants in the male ACL rupture subgroup. (D) Frequency distribution of the inferred haplotypes constructed from the TNC rs1061494 and rs2104772 variants in the male ACL rupture subgroup. CON: Control participants; ACL: Cases with ACL ruptures; NON: Cases reporting a non-contact mechanism of injury. Statistically significant differences in haplotype frequency between the groups are depicted on the graph, with p-values adjusted for age and weight and sex when all participants were evaluated. The number of participants (n) in each group is in parentheses. (TIF) [file pone.0205860.s001.tif]

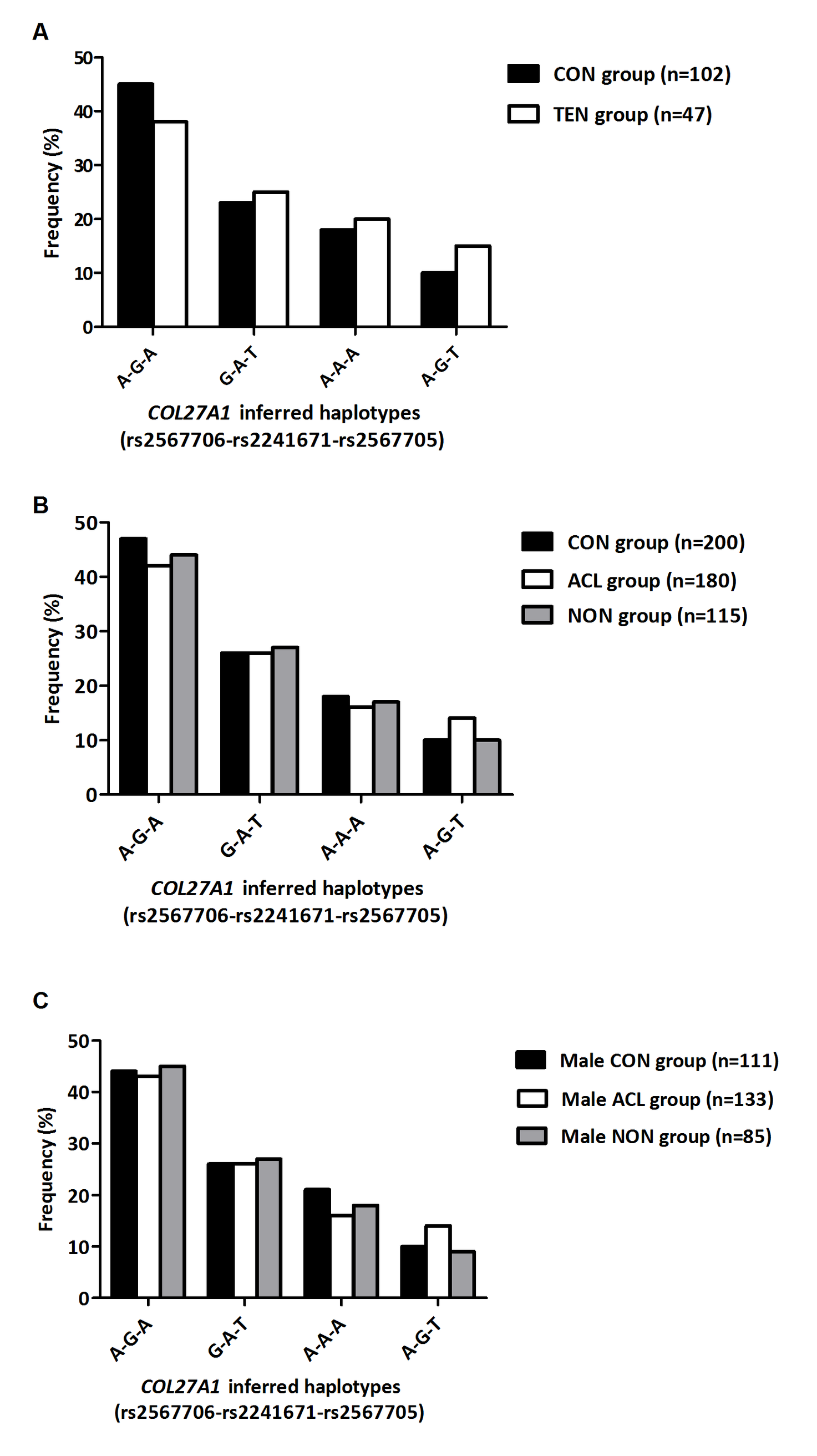

Supplement: S2 File — Frequency distribution of the inferred haplotypes constructed from the COL27A1 rs2567706, rs2241671 and rs2567705 variants in the (A) Achilles tendinopathy and (B) ACL rupture sample groups, in addition to the (C) male ACL rupture subgroup. CON: Control participants; ACL: Cases with ACL ruptures; NON: Cases reporting a non-contact mechanism of injury. Statistically significant differences in haplotype frequency between the groups are depicted on the graph, with p-values adjusted for age and sex in the Achilles tendinopathy sample group and age, sex and weight in the ACL rupture sample group. The number of participants (n) in each group is in parentheses. (TIF) [file pone.0205860.s002.tif]
