# Supplementary material for: Defining the molecular signatures of Achilles tendinopathy and anterior cruciate ligament ruptures: A whole-exome sequencing approach
Source: PLoS One. 2018 Oct 25;13(10):e0205860. doi: 10.1371/journal.pone.0205860 (PMC6201890; doi:10.1371/journal.pone.0205860)
Supplement: S1 Table — Values expressed as p-values. Effects on weight and BMI are adjusted for age and sex. Effects on height are adjusted for sex. P-values in bold typeset indicate significance (p<0.05). TEN: Achilles tendinopathy sample group. ACL: Anterior cruciate ligament rupture sample group. (DOCX) [file pone.0205860.s003.docx]

|  | **rs1061494** | | |  |  | **rs2104772** | | |  |
| --- | --- | --- | --- | --- | --- | --- | --- | --- | --- |
|  | **TEN** |  | **ACL** |  |  | **TEN** |  | **ACL** |  |
| Age (years) | 0.710 |  | 0.767 |  |  | 0.542 |  | 0.241 |  |
| Sex (% Males) | 0.564 |  | 0.153 |  |  | 0.237 |  | 0.757 |  |
| Mass (kg) | 0.429 |  | 0.158 |  |  | 0.255 |  | 0.277 |  |
| Height (cm) | 0.922 |  | 0.227 |  |  | 0.348 |  | 0.961 |  |
| BMI (kg/m^2^) | 0.266 |  | 0.551 |  |  | 0.159 |  | 0.197 |  |
|  | **rs1138545** | | |  |  | **rs1061495** | | |  |
|  | **TEN** |  | **ACL** |  |  | **TEN** |  | **ACL** |  |
| Age (years) | 0.465 |  | 0.403 |  |  | 0.217 |  | 0.231 |  |
| Sex (% Males) | 0.238 |  | 0.462 |  |  | 0.489 |  | 0.884 |  |
| Mass (kg) | 0.581 |  | 0.881 |  |  | 0.314 |  | 0.482 |  |
| Height (cm) | 0.450 |  | 0.993 |  |  | 0.622 |  | 0.855 |  |
| BMI (kg/m^2^) | 0.243 |  | 0.098 |  |  | 0.468 |  | 0.592 |  |
