# Supplementary material for: Defining the molecular signatures of Achilles tendinopathy and anterior cruciate ligament ruptures: A whole-exome sequencing approach
Source: PLoS One. 2018 Oct 25;13(10):e0205860. doi: 10.1371/journal.pone.0205860 (PMC6201890; doi:10.1371/journal.pone.0205860)
Supplement: S3 Table — P-values are for difference in genotypes and minor allele frequencies between diagnostic groups adjusted for age and weight at the time of recruitment for controls and the time of injury for cases, in addition to sex. Bold typeset indicates significance (p<0.05). Participant count for genotypes presented in parenthesis and total count in italics. a rs1138545 CC genotype vs. CT + TT genotype. b rs1061495 TT genotype vs. CT + CC genotype. (DOCX) [file pone.0205860.s005.docx]

| **ACL rupture group** | | | | | |
| --- | --- | --- | --- | --- | --- |
|  | CON | ACL | *p*-values | NON | *p*-values |
|  |  |  |  |  |  |
| rs1061494 | *(n=216)* | *(n =198)* |  | *(n=125)* |  |
| CC | 17 (36) | 16 (32) | 0.917 | 15 (19) | 0.598 |
| CT | 49 (106) | 53 (105) |  | 50 (62) |  |
| TT | 34 (74) | 31 (61) |  | 35 (44) |  |
| C Allele | 41 (178) | 43 (169) | 0.666 | 40 (100) | 0.807 |
| HWE | 0.891 | 0.105 |  | 0.720 |  |
|  |  |  |  |  |  |
| rs1138545 | (*n*=197) | (*n*=180) |  | (*n*=112) |  |
| CC | 74 (146) | 71 (127) | 0.611 | 69 (77) | 0.699 |
| CT | 21 (41) | 27 (48) |  | 29 (32) |  |
| TT ^a^ | 5 (10) | 3 (5) |  | 3 (3) |  |
| T Allele | 11 (43) | 16 (58) | 0.850 | 17 (38) | 0.668 |
| HWE | 0.014 | 0.804 |  | 1.000 |  |
|  |  |  |  |  |  |
| rs2104772 | (n=221) | (n=199) |  | (n=126) |  |
| AA | 25 (56) | 25 (50) | 0.846 | 25 (31) | 0.816 |
| AT | 44 (96) | 43 (85) |  | 44 (56) |  |
| TT | 31 (69) | 32 (64) |  | 31 (39) |  |
| A allele | 47 (208) | 46 (185) | 0.897 | 47 (118) | 1.000 |
| HWE | 0.111 | 0.356 |  | 0.389 |  |
|  |  |  |  |  |  |
| rs1061495 | *(n=221)* | *(n=198)* |  | *(n=126)* |  |
| CC ^b^ | 8 (18) | 7 (13) | 0.627 | 7 (9) | 0.308 |
| CT | 39 (85) | 33 (66) |  | 28 (35) |  |
| TT | 53 (118) | 60 (119) |  | 65 (82) |  |
| C Allele | 27 (121) | 23 (92) | 0.188 | 21 (53) | 0.080 |
| HWE | 0.618 | 0.460 |  | 0.124 |  |
