# Supplementary material for: Defining the molecular signatures of Achilles tendinopathy and anterior cruciate ligament ruptures: A whole-exome sequencing approach
Source: PLoS One. 2018 Oct 25;13(10):e0205860. doi: 10.1371/journal.pone.0205860 (PMC6201890; doi:10.1371/journal.pone.0205860)
Supplement: S4 Table — Values expressed as p-values. Effects on weight and BMI are adjusted for age and sex. Effects on height are adjusted for sex. P-values in bold typeset indicate significance (p<0.05). TEN: Achilles tendinopathy sample group. ACL: Anterior cruciate ligament rupture sample group. (DOCX) [file pone.0205860.s006.docx]

|  | **rs2567706** | | | |  | **rs2241671** | | | |  | **rs2567705** | | |
| --- | --- | --- | --- | --- | --- | --- | --- | --- | --- | --- | --- | --- | --- |
|  | **TEN** |  | **ACL** |  |  | **TEN** |  | **ACL** |  |  | **TEN** |  | **ACL** |
| Age (years) | 0.818 |  | 0.655 |  |  | 0.863 |  | 0.271 |  |  | 0.440 |  | 0.662 |
| Sex (% Males) | 0.917 |  | 0.166 |  |  | 0.814 |  | 0.773 |  |  | 0.853 |  | 0.508 |
| Mass (kg) | 0.732 |  | 0.306 |  |  | 0.499 |  | 0.630 |  |  | 0.334 |  | 0.705 |
| Height (cm) | 0.542 |  | 0.975 |  |  | 0.337 |  | 0.227 |  |  | 0.076 |  | 0.624 |
| BMI (kg/m^2^) | 0.562 |  | 0.682 |  |  | 0.243 |  | 0.477 |  |  | 0.457 |  | 0.266 |
